# Interlayer Bound Wannier Excitons in Germanium Sulfide

**DOI:** 10.3390/ma13163568

**Published:** 2020-08-12

**Authors:** Sara Postorino, Jianbo Sun, Saskia Fiedler, Laurent O. Lee Cheong Lem, Maurizia Palummo, Luca Camilli

**Affiliations:** 1 Dipartimento di Fisica, Università degli studi di Roma “Tor Vergata”, via della Ricerca Scientifica 1, 00133 Roma, Italy; sara.postorino@uniroma2.it; 2 Department of Physics, Technical University of Denmark, Ørsteds Plads, 2800 Kgs. Lyngby, Denmark; jisun@dtu.dk; 3 Centre for Nano Optics, University of Southern Denmark, Campusvej 55, 5230 Odense M, Denmark; safi@mci.sdu.dk; 4 Australian National Fabrication Facility, Australian National University, Canberra 2601, ACT, Australia; u1077231@anu.edu.au; 5 Istituto Nazionale di Fisica Nucleare, via della Ricerca Scientifica 1, 00133 Roma, Italy

**Keywords:** germanium sulfide, cathodoluminescence, 2D materials, density functional theory, anisotropy, many body perturbation theory, hexagonal boron nitride

## Abstract

We report a cathodoluminescence (CL) study of layered germanium sulfide (GeS) where we observe a sharp emission peak from flakes covered with a thin hexagonal boron nitride film. GeS is a material that has recently attracted considerable interest due to its emission in the visible region and its strong anisotropy. The measured CL peak is at ~1.69 eV for samples ranging in thickness from 97 nm to 45 nm, where quantum-confinement effects can be excluded. By performing *ab initio* ground- and excited-state simulations for the bulk compound, we show that the measured optical peak can be unambiguously explained by radiative recombination of the first free bright bound exciton, which is due to a mixing of direct transitions near the Γ-point of the Brillouin Zone and it is associated to a very large optical anisotropy. The analysis of the corresponding excitonic wave function shows a Wannier–Mott interlayer character, being spread not only in-plane but also out-of-plane.

## 1. Introduction

Over the past two decades, a wide range of layered/2D materials has been under extensive experimental and theoretical scrutiny because of their stability, interesting chemical and physical properties, the possibility to be exfoliated or grown in ultra-thin films, and the potentiality to realize flexible electronic and opto-electronic devices [1,2,3,4,5,6,7,8,9,10]. Among them, the family of group-IV monochalcogenides recently started to raise interest both at experimental and theoretical level. Indeed owing to their large in-plane anisotropy, they offer one more dimension to manipulate the physical properties when compared with isotropic layered/2D materials, making them particularly attractive to realize novel angle-dependent electronic, opto-electronic [11], and tunable spintronic devices [12]. Regarding the optical properties, the most representative material of this family is germanium monosulfide (GeS).

GeS crystallizes in a low-symmetry structure similar to that of black phosphorous (BP) [13]. Like BP, it is also sensitive to oxygen and thus tends to oxidize if left in air [14,15]. From a technological point of view, GeS is an interesting material because it has an optical band gap in the visible range and exhibits high photosensitivity and broad spectral response [16,17,18]. First-principle calculations indicate that the bandgap of GeS can be effectively tuned by applying an external strain, which allows the modulation of the emission wavelength [19,20,21]. GeS also exhibits giant piezoelectricity due to its characteristic “puckered” C2v symmetry [22]. Additionally, a study by molecular dynamics simulations predicts that monolayer GeS also exhibits an electrocaloric effect [23]. Moreover, its strong out-of-plane and in-plane optical anisotropy makes the realization of polarization-sensitive photodetectors and angle-dependent optoelectronic devices possible [20,24,25,26,27].

In this article, we investigate the optical properties of hBN-coated GeS by using cathodoluminescence (CL) measurements and *ab initio* calculations based on density function theory (DFT) and Many-body Perturbation Theory (MBPT). Notably, we find a sharp peak at 1.69 eV that can be assigned to the radiative recombination of intrinsic direct bound excitons due to transitions near the Γ point. Our study illustrates the optical emission characteristics of GeS, which is instructive for the exploration of its applications in diverse fields.

## 2. Experimental and Theoretical Methods

The GeS flakes are exfoliated from a bulk crystal (ordered from 2D Semiconductors) using thermal release tape (EPL BT-150E-KL) and then deposited on a p-type Si wafer. Briefly, after exfoliating the crystal for multiple times with the tape, the flake-loaded tape is applied onto the Si substrate and then removed after being heated at 75 ${}^{\circ }$C for 5 min on a hotplate. The thin flakes are then identified using an optical microscope (Nikon L200ND) and the selected flakes showing areas of multiple thicknesses are then coated with hBN (∼20 nm thick, HQgraphene, previously prepared in the same way as that for GeS flakes).

The thickness of the flakes is evaluated from AFM measurements carried out with a NTEGRA scanning probe microscope (NT-MDT Spectrum Instruments). The scans are performed in semi-contact mode with standard Si cantilevers.

The Raman spectra are taken with a Thermo Fisher DXR Raman spectrometer using a 455 nm laser with a power of 0.5 mW under a 100× objective lens. For the angle-resolved measurements, the samples are rotated using a rotation stage, which gives an error of $\pm 2^{\circ }$.

CL spectroscopy is performed in a FEI Verios 460 with a Gatan MonoCL 4 Elite. The CL spectra are collected with acceleration voltage of 5 kV and beam current of 1.6 nA after correction for dark counts. All the CL spectra are measured at room temperature.

The independent-particle results discussed in this paper are obtained performing DFT simulations using the plane-wave Quantum-Espresso code [28]. A Perdew–Burke–Ernzerhof (PBE) exchange-correlation functional (PBE [29]) with Grimme-d2 VdW correction [30] to take into account the interaction between the layers is used. Scalar relativistic optimized norm-conserving [31] pseudopotentials from the QE repository are used. To evaluate the role of spin–orbit interaction, selected calculations are done using the fully relativistic version of the same pseudopotentials from the same repository. A 14×16×4 Monkhorst–Pack grid [32] of *k*-points to sample the Brillouin zone and a kinetic energy cut-off of 80 Ry are used for structural optimization runs. Structure relaxation is assumed at convergence when the maximum component of the residual forces on the ions is smaller than 10${}^{-4}$ Ry/Bohr.

Once the optimized atomic structure is obtained, self and non-self consistent DFT calculations are performed to obtain Kohn–Sham (KS) eigenvalues and eigenfunctions to be used in the many-body simulations done by using the YAMBO code [33]. Specifically, we calculate the quasi-particle (QP) energies by using the $GW_{0}$ perturbative method [34], while the optical excitation energies and optical spectra are obtained by solving the Bethe–Salpeter Equation (BSE) [35,36,37,38,39]. For GW simulations a plasmon-pole approximation for the inverse dielectric matrix is applied [40], kinetic energy cut-off of 16 Ry (160 Ry) is used for the correlation, $\Sigma_{c}$ (exchange, $\Sigma_{x}$), part of the self-energy and the sum over the unoccupied states for $\Sigma_{c}$ and the dielectric matrix is performed up to ∼50 eV above the VBM. In order to speed up the convergence with respect to empty states we adopt the technique described in [41].

The Bethe–Salpeter Equation (BSE) used to obtain the optical spectrum, the exciton energy, as well as its spatial localization is solved within the Tamm–Dancoff [36,42] approximation (which is generally valid for bulk compounds to describe neutral excitations well below the plasma frequency of the material). We use four occupied and four unoccupied states to build up the excitonic Hamiltonian to obtain a good description of the spectrum up to ~5 eV.

The convergence with the *k*-sampling for the BSE calculations has been checked and we use a 22×24×2 uniform grid centered in Γ. To plot the excitonic wavefunction along the YZ plane we use a 8×10×4 grid, in order to avoid spurious replica effects along the Z direction, after verifying that the low energy part of the spectrum is well converged using both *k*-grid samplings.

## 3. Results and Discussion

**Figure 1:**
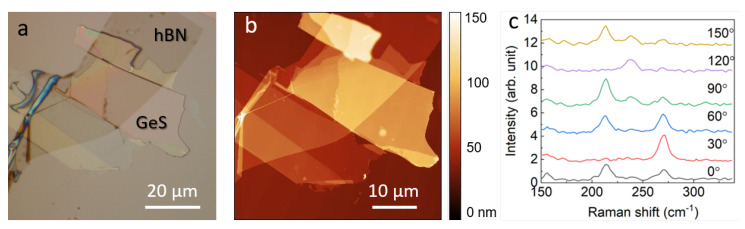
(**a**) Optical image and (**b**) AFM image of the hBN-coated GeS flake showing areas of different thickness. (**c**) Typical angle-resolved Raman spectra of the GeS flake. The relative intensity of the GeS Raman peaks changes as a function of the angle (0–150 degree) between the incident polarized light and the flake.

An optical image of the studied GeS flake that is deposited on a Si substrate and covered by a thin hBN film is displayed in Figure 1a. The flake contains regions of different thickness, as can be seen from the AFM image in Figure 1b, with the thickness ranging from 12 to 97 nm. We also characterized the GeS samples with Raman spectroscopy, obtaining the characteristic three main Raman peaks at 213, 240, and 270 cm${}^{-1}$, which are assigned to $B_{3g}$, $A_{g}^{1}$, and $A_{g}^{2}$ symmetry, respectively (Figure 1c). As expected because of the anisotropic nature of GeS, the intensity of the Raman scattering peaks depends strongly on the polarization of the incident light (Figure 1c) [24,43].

**Figure 2:**
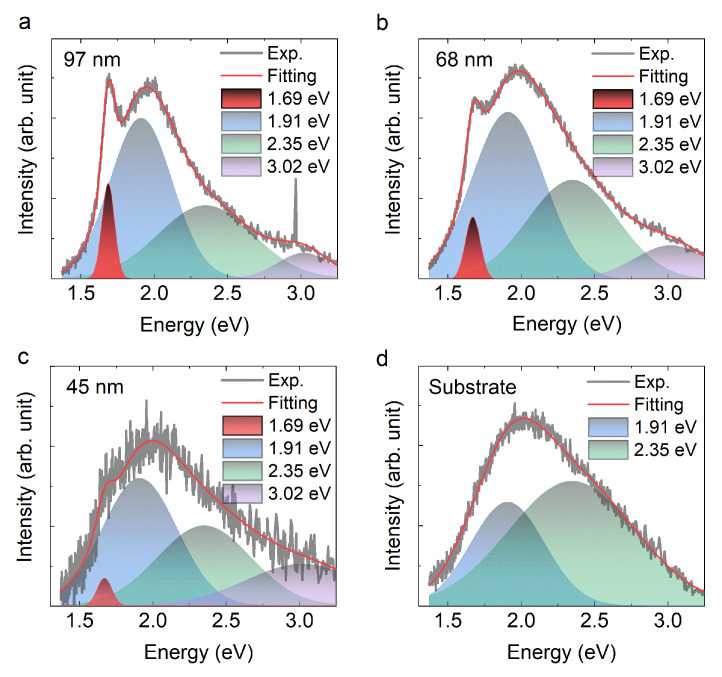
(**a**–**c**) Cathodoluminescence (CL) spectra of the GeS flakes with thickness of 97 nm, 68 nm, and 45 nm; all these spectra can be deconvoluted with four Gaussian components with centers at 1.69, 1.91, 2.35, and 3.02 eV. (**d**) The substrate CL spectrum can be fitted with only two Gaussian components centered at 1.91 and 2.35 eV.

Representative CL spectra collected from three areas of the GeS flake, with thicknesses of 97, 68, and 45 nm, are displayed in Figure 2a–c. The spectra can be deconvoluted into four Gaussian components with centers at 1.69, 1.91, 2.35, and 3.02 eV, respectively. The Gaussian components at 1.91 and 2.35 eV originate from the substrate, as determined by analyzing the spectrum taken from an area outside the GeS flake (Figure 2d). The intrinsic emission from GeS is at 1.69 eV, in agreement with data obtained from photoluminescence (PL) experiments [24,43]. In the following paragraphs, we show that this peak is attributed to the radiative recombination of intrinsic direct bound excitons near Γ point in the Brillouin Zone (BZ) of GeS. Interestingly, while in the spectrum collected on the bare substrate in an area away from the flakes the intensity of the peak at 2.35 eV is higher than that of the peak at 1.91 eV (Figure 2d), their relative ratio is reversed in the spectra collected from the GeS flakes (Figure 2a–c). We ascribe this difference to the absorption by GeS of the signal at 1.91 eV. This behavior is better explained later on, when we take into account the theoretical results. The small peak centered at 3.02 eV is attributed to the hBN film; most likely it is due to the second-order diffraction of the band edge emission by the gating that is not filtered in the set-up. However, we cannot entirely exclude that it might be a defect-related emission [44], although rather unlikely due to the broad full width at half maximum of the peak. Additionally, it is worth mentioning that for thinner flakes (below 45 nm), the signal-to-noise ratio was too low and no obvious peak at 1.69 eV could be observed with our current setup.

**Figure 3:**
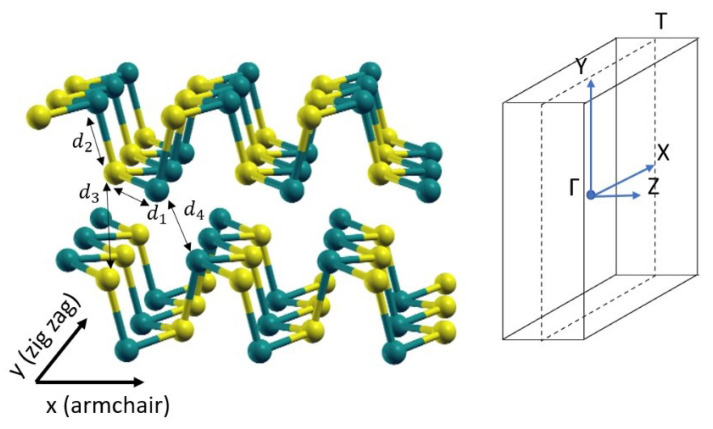
GeS crystal structure (Ge yellow, S blue) on the left and its Brillouin Zone on the right.

In order to perform all the calculations, the initial atomic structure of GeS in the orthorombic phase is directly taken from the Materials project website [45]. Then, we carry out a full relaxation of the cell parameters and atomic positions. In Figure 3, the final relaxed atomic structure is shown, while in Table 1 we report the numerical values of main geometrical parameters (lattice constants a,c and interatomic distances $d_{1}-d_{4}$ as shown in Figure 3), and compare them with available experimental and theoretical data [46].

**Figure 4:**
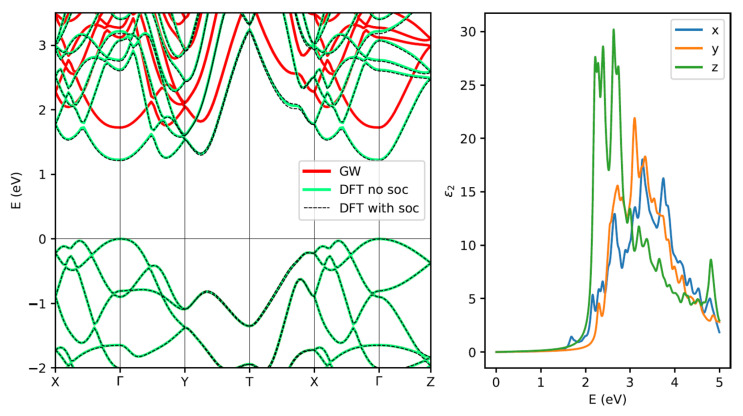
**Left panel**: Bulk GeS DFT bandstructure neglecting relativistic effects (green, solid) and taking them into account (black, dashed) and its quasiparticle bandstructure (red, solid). **Right panel**: Imaginary part of the dielectric functions calculated at the GW+BSE method, with light polarized along X, Y, and Z directions.

Once the equilibrium geometry is obtained, we calculate, at the same level of theoretical approximation, the electronic band structure along the main high-symmetry directions of the BZ with (green) and without (black) taking into account of the spin–orbit coupling, as reported in Figure 4 (left panel). It is evident that the relativistic correction does not affect in a significant way the band structure and, for this reason, we neglect it in all the many-body simulations. In agreement with previous literature [47], the minimum gap is direct at Γ point of the BZ, although another point in the ΓY direction is almost degenerate. The value of the DFT direct gap is 1.23 eV, which is clearly underestimated due to the well-known band-gap problem of DFT-KS approach in any local or semi-local xc-flavour. For this reason, we report in the same figure also the corresponding quasi-particle band structure (red curve), calculated within the $GW_{0}$ perturbative approach. At this level of theoretical approximation the minimum direct gap at Γ increases to 1.78 eV, while the dispersion remains essentially unchanged.

In Figure 4 (right panel) we report the imaginary part of the three components of the dielectric function for light polarized along the arm-chair (X) and zig-zag (Y) directions and perperdicularly (Z) to the atomic planes. They are calculated taking into account both local-field and excitonic effects by solving the Bethe–Salpeter Equation (BSE). The first optical peak at ~1.68 eV is due to a mix of single quasi-particle transitions from valence band maximum (VBM) to conduction band minimum (CBM) that involve Γ and few *k*-points around it. A strong optical anisotropy is observed, as can be seen from the presence of an excitonic peak at low energies only for light-polarized along the arm-chair direction. This is well explained by the analysis of the states involved in the excitonic transition, as discussed later.

**Figure 5:**
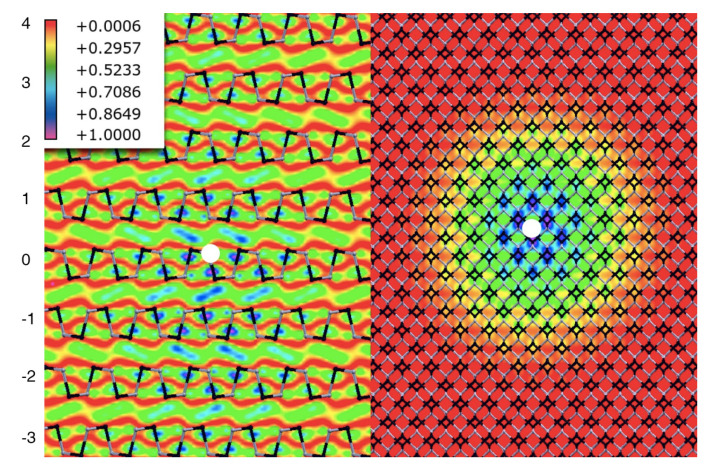
Plot of the square modulus of the first bright excitonic wavefuction, fixing the hole position near an S atom, represented by the white dot at the center. Side view on the left, top view on the right. Numbers indicates the layers with respect to the one with the hole.

Figure 5 shows the spatial distribution (left panel: side view; right panel: top view) of the first bright exciton, plotting the square modulus of the excitonic wavefunction at a fixed hole position (near a S atom, represented by the white dot). Interestingly the exciton has a strong interlayer character being very delocalized not only in-plane, with a Bohr radius of about 2 nm (right panel), but also out-of-plane. In other words, the probability of finding the electron far from the hole position is high across several layers above and below the layer where the hole is created (see the violet/blue parts of the isosurface in the left panel). It is worth to mention here that the Wannier-like character of the first bright exciton is consistent with previous studies, done for the case of GeS monolayer [48] and 2D [49] heterostructures, by using the same theoretical approach. From the values of the minimum electronic quasi-particle gap and of the first bright exciton energy, we estimate an exciton binding energy of 0.1 eV which is 10 times larger of the value reported in [50] by Tuttle et al. using a similar theoretical approach and where an underestimation of ~0.2 eV of both the electronic and optical gaps is also found. It is worth also to mention that the estimated exciton binding energy is of the same order, even slightly larger, with respect to those found for group-VI TMD bulk materials, where values ranging from 0.04 eV to 0.08 eV are reported [51,52].

**Figure 6:**
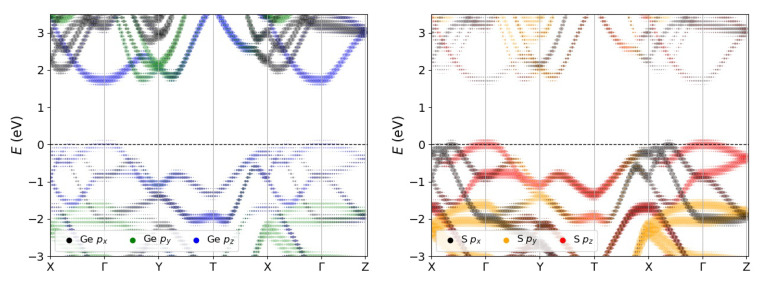
GW bands projected on atomic orbital bands. **Left**: Ge orbitals; **Right**: S orbitals.

**Figure 7:**
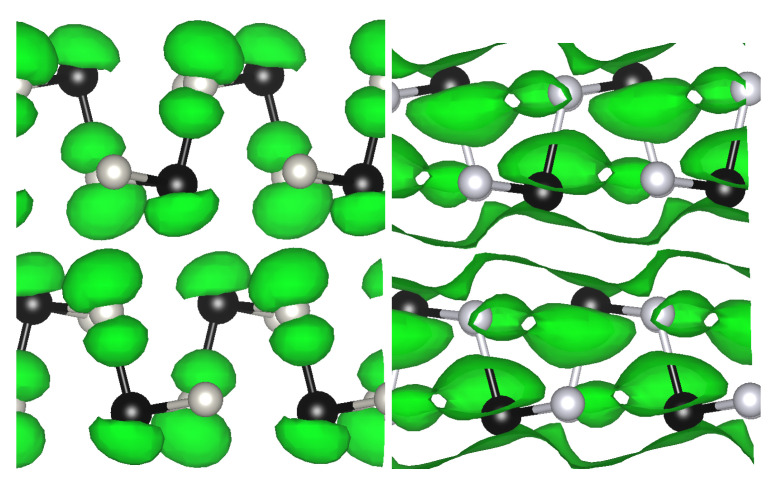
Plot of ${\left|\psi \right|}^{2}$ for $\Gamma_{v}$ (**left**) and $\Gamma_{c}$ (**right**). In green the isosurfaces at 1% of maximum value. The represented atoms are: Ge (black), S (gray).

To understand the anisotropy of the optical spectra we investigate the role and the spatial distribution of the atomic orbitals involved in the first optical peaks. In the two panels of Figure 6, we show an analysis of the character of the states in terms of atomic orbitals by using a *k*-resolved Projected Density of States (PDOS) calculation. The most important contributions to the bands, near the Fermi level, comes from *p* orbitals of Ge and S atoms. The highest occupied states are mainly due to *p* orbitals of S, while the ones of Ge are prevalent in the lowest unoccupied states. The *d* orbitals of Ge contribute to bands very low in energy which are not shown in the figure. In particular, near the Γ point we can see that the dominant contribution to the VBM (CBM) comes from $p_{z}$ orbitals of S-atoms (Ge-atoms) with a small contributions of $p_{x}$ orbitals of Ge (S). To further elucidate the character of the orbitals at the band edges, we report in Figure 7 the plot of the square modulus of the VBM and CBM wavefunction at Γ. A clear extension of the orbitals along the X direction (arm-chair) is visible both in valence (left panel) and even more in conduction (right panel) band. This explains the large optical anisotropy observed (see Figure 4), where in the low energy side, around 1.68 eV, only optical transitions for light polarized in the *X* direction (arm-chair) are allowed. The first peak (at ~2.25 eV) for light polarized along *Y* (zig-zag) is mainly due to *k*-points in the first half of the ΓY direction and involves transitions from the last occupied to the first unoccupied band. The large anisotropy is again easily explained by the dominant contribution of $p_{z}$ and $p_{y}$ orbitals (see Figure 6). Similarly the strong optical peaks for light polarized along *Z*, below and above 2.5 eV, involve the same bands but are due to *k*-points in the first half of ΓY and XT directions respectively, where a dominant contribution of $p_{z}$ orbitals is present. The analysis of the *k*-resolved PDOS allows also to explain why when GeS is thinned to a few layers, it becomes an indirect semiconductor [47]. Indeed, the valleys between Γ and Y are found for thin samples to be lower in energy and this is due to the fact that the CBM at Γ is pushed higher in energy with respect to the other valleys due to its main $p_{z}$ character.

**Figure 8:**
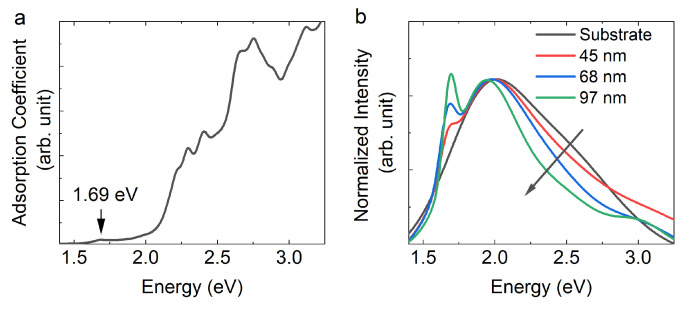
(**a**) The calculated absorption coefficient of bulk GeS, obtained averaging over the three components of the dielectric tensor. (**b**) The CL spectra reported in Figure 2 after normalization with respect to the maximum intensity of the broad peak at ~2 eV.

After the introduction of the theoretical optical spectra, it is interesting to discuss again the results presented in Figure 2. In this figure, we noticed an inversion of the intensity ratio of the peaks at 1.91 and 2.35 eV when the signal is collected on the bare substrate and on the GeS flake. This behavior is well explained by the calculated absorption spectrum of bulk GeS as displayed in Figure 8a. Indeed, it can be seen that the absorption coefficient at ~2.35 eV is around one order of magnitude higher than that at ~1.91 eV. This also explains why the intensity of the CL data for energies higher than 2 eV decreases as the thickness of GeS flakes increases, as shown in Figure 8b. The GeS flake is absorbing the light emitted from the substrate.

## 4. Conclusions

To conclude, we investigated the optical properties of multilayered GeS using cathodoluminescence at room temperature. A sharp emission peak is observed at ~1.69 eV, which is attributed to the radiative recombination of the first free bright direct exciton near the Γ-point of the Brillouin Zone, obtained from the *ab initio* ground and excited-state simulations of GeS bulk. An exciton binding energy of about 0.1 eV is estimated from the position of the first optical peak and the electronic minimum gap calculated within the GW approach. We find that the wave function of the first bright exciton is highly delocalized, spreading in the atomic plane and over several atomic layers. This is a quite peculiar behavior with respect to other layered materials, such as TMDs, where low energy excitons are generally localized in one or two atomic layers [53,54]. It also suggests a large dependence from quantum-confinement as well as the possibility, due to the e–h spatial extension, to further modulate the optical properties of multilayer GeS flakes by applying an external electric field perpendicularly to the atomic layers.

## Figures and Tables

**Table 1:** Lattice parameters and distances calculated at density function theory (DFT) level taking into account VdW interaction compared with experimental results [46] and previous theoretical results [47].

**Lattice Parameters**	**PBE-VdW (Å)**	**PBE (Å) [47]**	**Experiment (Å) [46]**
a	4.366	4.40	4.299
b	3.666	3.68	3.646
c	10.679	10.81	10.481
**Distances**	**PBE-VdW (Å)**	**Experiment (Å)** [46]	
$d_{1}\left(Ge-S\right)$	2.450	2.438	
$d_{2}\left(Ge-S\right)$	2.439	2.448	
$d_{3}\left(Ge-Ge\right)$	3.461	3.329	
$d_{4}\left(S-S\right)$	3.694	3.644	
